# A bibliometric analysis and review of recent researches on Piezo (2010-2020)

**DOI:** 10.1080/19336950.2021.1893453

**Published:** 2021-03-16

**Authors:** Lifu Wang, Xuening Liu, Kun Zhang, Zhongcheng Liu, Qiong Yi, Jin Jiang, Yayi Xia

**Affiliations:** a The Second Clinical Medical College of Lanzhou University, Lanzhou, PR China; b Department of Orthopedics, Gansu Key Laboratory of Orthopaedics, Lanzhou University Second Hospital, Lanzhou Gansu, China

**Keywords:** Bibliometric, piezo, citespace, vosviewer

## Introduction

The Piezo channel protein is a type of mechanical-sensitive ion channel that was discovered by Coste et al. in 2010 using RNA interference technology in a mouse Neuro2A cell line [1]. Since its discovery, Piezo has attracted the attention of scholars in various fields. Many studies have reported that Piezo is necessary for cells to respond to mechanical stimuli and can convert mechanical signals sensed by the membrane into intracellular electrical or chemical signals. In vertebrates, Piezo channel proteins mainly include Piezo1 and Piezo2 proteins, which are encoded by the genes *FAM38A* and *FAM38B*, and consist of 2500 and 2800 amino acids, respectively. The Piezo1 protein is highly expressed in the lungs, bladder, and skin [2], whereas Piezo2 is mainly expressed in the nervous system [3]. In recent years, it has been found that Piezo1 can be activated by a variety of mechanical stresses, including compressive stress [4], stretching [5], and fluid shear stress [6].

In 2014, the Piezo protein was found to play a regulatory role in the mechanical signal transduction of chondrocytes. Lee [7], and other researchers, revealed that Piezo1 and Piezo2 were highly expressed in the mechanical environment of chondrocytes and that the apoptosis rate of chondrocytes could be reduced by treatment with the Piezo1 channel inhibitor GsMTx4. Servin-Vences et al. [8] found that Piezo1 can mediate a tension-induced intracellular current in chondrocytes using a high-speed pressure clamp method. After silencing Piezo1 expression in chondrocytes with miRNA, the number of chondrocytes responding to mechanical stimulation decreased significantly (50%), while chondrocytes treated with the Piezo1 activator (Yoda1) exhibited a significantly enhanced mechanical response, which further confirmed the role of Piezo1 in mechanical stress signal transduction in chondrocytes. In 2017, Sugimoto et al. [9] reported that Piezo1 can promote osteogenic differentiation of bone marrow mesenchymal stem cells under compressive stress. The Piezo channel also plays an important role in hemopathy. In 2012, Zarychanski et al. found that mutations in the mechanotransduction protein (PIEZO1) contribute to hereditary xerocytosis [10]. The Piezo 1 channel was also shown to be required for vascular development in mice [11]. These studies revealed that the Piezo channel plays an important role in human health. As research in the Piezo channel continues to expand, the literature continues to progress as well. However, the topics and characteristics of most-cited articles on the Piezo channel have yet to be investigated.

Bibliometric techniques are statistical analysis and quantitative tools that have been used in information research and library practice over a long period [12]. In addition, bibliometric methods can help researchers understand how the knowledge within a publication is used by a process involving the extraction of measurable data through statistical analysis of published research [13]. By using statistical and quantitative tools, bibliometric analysis can demonstrate information of a certain field to researchers, including patterns of countries, institutions, journals, authors, and keywords related to specific publication types [14].

In this study, we aimed to provide a report on scientific production in research on the Piezo channel among countries over the last 10 years (2010 − 2020), apply bibliometry to dissect the characteristics of scientific articles in the Web of Science (Thomson Reuters Company) into several components so that the subjective factors are minimalized, and analyze the overall publication trends related to research on the Piezo channel.

## Methods

### Data source and search strategy

The Science Citation Index-Expanded (SCI-E) of the Web of Science Core Collection (WoSCC) was used to perform this cross-sectional study. Queries using varying Boolean searches were conducted in January 2021 and the searches were all completed in a single day (3 January 2020) to avoid bias caused by the daily updates of the database. The search strategy was as follows: TS = (Piezo1 OR Piezo 2 OR FAM38A) AND LANGUAGE: (English) AND DOCUMENT TYPES: (ARTICLE OR PROCEEDINGS PAPER).

**Figure 1.**
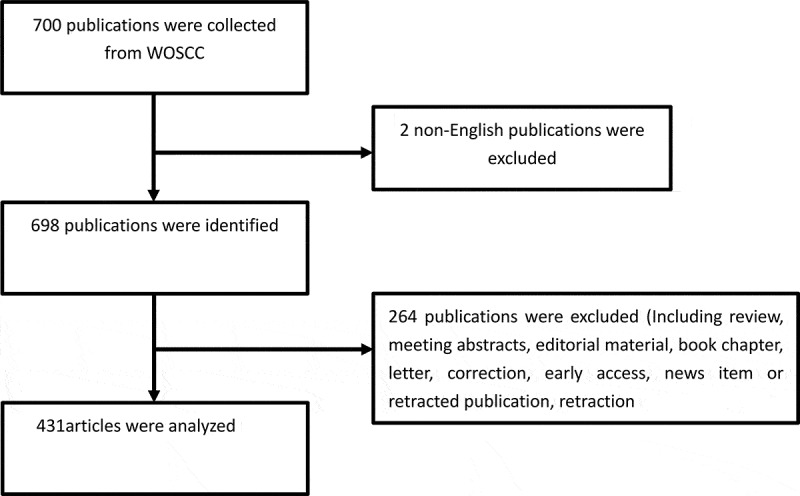
Flowchart of Piezo research

In total, 700 publications were obtained, and the following categories were excluded: review articles, meeting abstracts, editorial material, book chapters, letters, corrections, early access, news items or retracted publications, and retractions. In total, 431 articles were analyzed. The retrieval strategy of the experiments is shown in Figure 1.

### Data collection

Initially, the records retrieved from WoS were downloaded, screened, sorted, and extracted. Then, these data sets were converted to txt format and imported into CiteSpace V 5.7.R2, 64bit, and VOSviewer 1.6.11.

### Statistical analysis

We reviewed the characteristics of publications, including the distribution of countries/regions, institutions, journals and authors, number of annual publications, citation counts, and H-index, by establishing the WoS Literature Analysis Report online. The “H-index” is used as a tool in predicting future research. For example, if a researcher’s “H-index” is 20, it means that this researcher has a total of 20 papers that have been cited at least 20 times. The consequence and number of co-cited authors and co-cited references were calculated using VOSviewer (Leiden University, Leiden, Netherlands).

## Results

### General information and annual publication output

**Figure 2.**
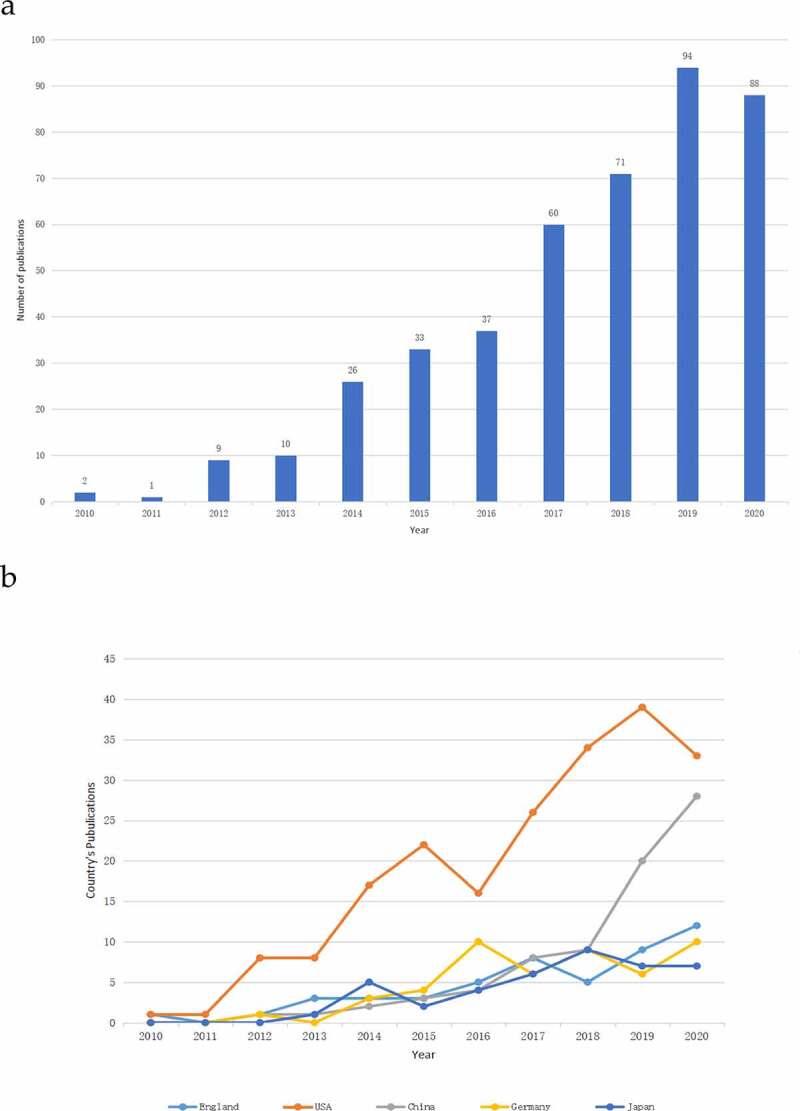
Publication outputs and growth trend. (a) The number of annual publications on Piezo research from 2010 to 2020; and (b) The line chart of publication trend related to Piezo among different countries

A total of 700 articles were retrieved. To explore the trends of research on the Piezo channels, we presented the number of articles per year in the form of a histogram, which showed that the number of publications related to the Piezo channel has generally increased. Moreover, the overall trend increased from two papers in 2010 to 94 papers in 2019, with the number of published articles peaking in 2019 (Figure 2a). Overall, the publication output in the top five countries continued to grow between 2010 and 2020, with the exception of in the United States (Figure 2b).

### Citation and H-index analysis

**Figure 3.**
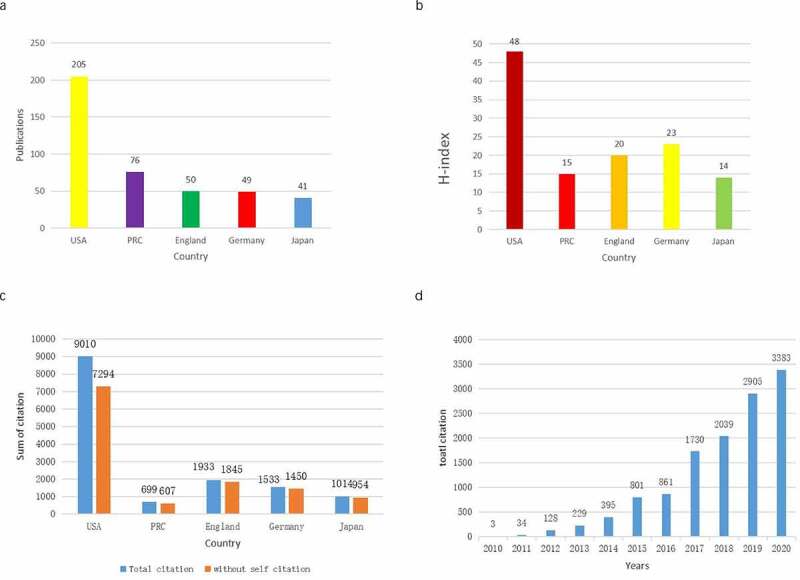
The publications (a), H-index (b), and citation counts (c) in the top 5 countries; and (d) The number of citations related to Piezo research from 2010 to 2020

Through analysis of the WOS database, we identified that the United States published the most papers in the past 10 years, followed by China (Figure 3a). Papers from the United States had the highest number of citations (9010), accounting for 72.79% of the total citations. The H index of papers from the United States was 48. England ranked second with 1933 citations (15.45%), with an H index of 20. Germany followed, with a frequency of 1540 citations (12.31%) and an H-index of 23 (Figure 3b and 3c). Although China ranked second in the number of articles published (with a number of 76), the total number of citations (699) was the lowest in the top five countries and the H-index (15) was the second to last. This indicates that the quality and influence of articles from China also need to be improved. All Piezo-related publications included in this study were cited 12,508 times from 2010 to 2020. The average number of citations of each paper was 29.02. Figure 3d shows the number of total citations per year from 2010 to 2020. Over the past 10 years, the total number of citations in this field has continued to increase.

#### Active countries/regions and institutions

**Figure 4.**
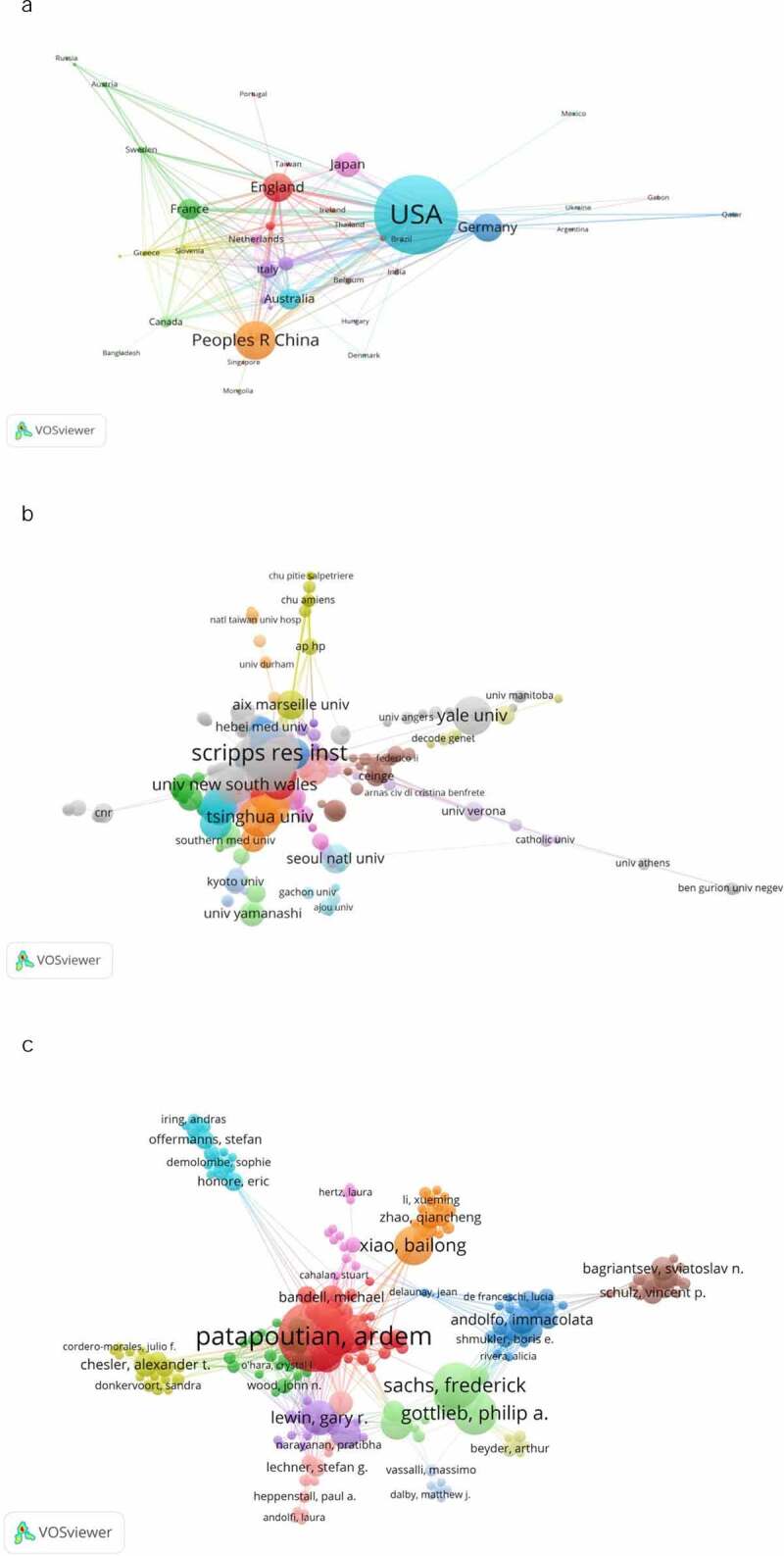
The network map. (a) The network map of countries/regions, (b) the network map of institutions, and (c) the network map of authors who conducted Piezo research

Researchers from 49 countries participated in Piezo research. The United States produced the most papers (205, 47.56%), followed by China (76, 17.63%) and England (50, 11.60%). The cooperative relationship among these countries is demonstrated in Figure 4a. As demonstrated in Figure 4a, the United States attached great importance to cooperation and had close collaborations with China, Germany, England, and Japan. The data contributed by different countries/regions and institutions are shown in Table 1. Most of the publications originated from institutions in the United States, with The Scripps Research Institute producing the highest number of publications on Piezo (27), followed by SUNY Buffalo (19) and Tsinghua University (14); the connection among these institutions is demonstrated in Figure 4b.

**Table 1.** The top 10 Institutions and Country/region contributed to the publications on Piezo researches

Rank	Institution	Frequency	Country/region	Frequency
1	Scripps Res Inst	27	USA^1^	205
2	SUNY^2^ Buffalo	19	Peoples R China	76
3	Tsinghua University	14	England	50
4	Novartis Res Fdn	13	Germany	49
5	Yale university	13	Japan	41
6	Duke university	12	France	32
7	The University of New South Wales	12	Australia	32
8	Max Delbruck Center for Molecular Medicine	11	Italy	24
9	Stanford University	11	Spain	19
10	University of Alabama at Birmingham	10	Canada	15

1. USA: United States of America;2. SUNY: State University of New York;

### Active authors

A total of 2,773 authors contributed 431 articles related to Piezo channel research. The network of authors contributing to Piezo research is demonstrated in Figure 4 c. Table 2 lists the top 10 most active and most cited authors. In the network of authors contributing to Piezo channel research, the largest node was Ardem Patapoutian, who was the first researcher to discover Piezo as a mechanically sensitive ion channel [1] and whose works laid the foundation for the major research projects being undertaken today. Frederick Sachs was the second most highly published author. His works focused on the regulation of the Piezo channel [15–19], and his most influential work focused on mutations in Piezo1 [20]. In addition, Ardem Patapoutian, from The Scripps Research Institute, which is known as the most influential institution in the world for its impact on innovation, was the most productive author and had the highest citation counts.

**Table 2.** The top 10 authors that published articles on Piezo researches and the top 10 cited authors

Rank	Author	Freq	Author	Citations
1	Patapoutian, Ardem	27	Patapoutian, Ardem	3917
2	Sachs, Frederick	16	Coste, Bertrand	2674
3	Gottlieb, Philip A.	15	Mathur, Jayanti	2418
4	Coste, Bertrand	13	Dubin, Adrienne E.	2126
5	Xiao, Bailong	13	Schmidt, Manuela	1417
6	Gu, Jianguo .	12	Woo, Seung-Hyun	1053
7	Lewin, Gary R.	11	Bandell, Michael	968
8	Martinac, Boris	10	Ranade, Sanjeev S.	838
9	Mathur, Jayanti	10	Sachs, Frederick	825
10	Dubin, Adrienne E.	9	Murthy, Swetha E.	761

### Active journals

The 431 articles were published in a total of 189 journals, with 19 journals having published more than five papers in this field, accounting for about 10.05% of the published literature. The top 10 journals in terms of the number of publications are shown in Table 3. Nature Communications (IF = 12.121) published 26 articles, which was the highest number, whereas Proceedings of the National Academy of Sciences of the United States of America (IF =** 9.412**) published 21 papers; Scientific Reports (IF = 3.998) ranked third with 21 publications.

**Table 3.** The top 11 journals that published articles on TRPM7 researches

Journal	Frequency	Countries/regions	IF*
Nature communications	26	England	12.121
Proceedings of The National Academy of Sciences of The United States of America	21	USA	9.412
Scientific Reports	21	England	3.998
Cell Reports	18	England	8.109
eLife	18	England	7.080
Nature	14	England	42.778
Plos One	10	USA	2.740
Neuron	9	USA	14.415
Neuroscience Letters	9	Netherlands	2.274
Frontiers In Physiology	8	SWITZERLAND	3.367

*IF: Impact Factor (2020)

**Figure 5.**
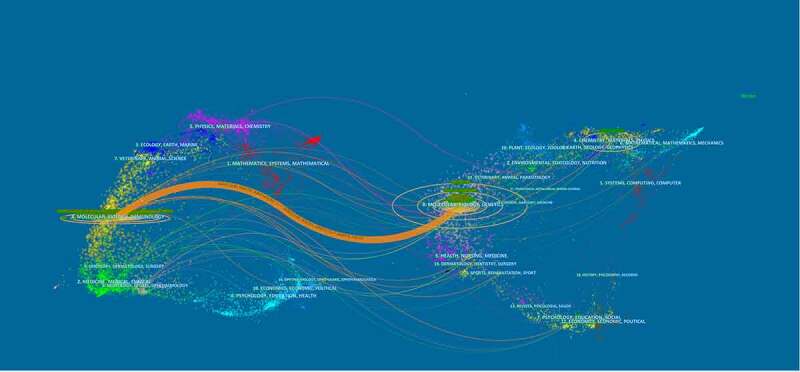
The dual-map overlay of journals related to Piezo research

In this dual-map, the left side presents the journals that have published papers on Piezo and the right side presents the journals from which the references originated. Figure 5 indicates that most papers on Piezo were published in the fields of molecular biology and immunology, and they cited journals in the areas of molecular biology, and genetics.

### Keyword co-occurrence and burst

Using CiteSpace, keywords were identified and analyzed via the strong citation bursts (Table 4). The keywords with strong bursts after 2010 were as follows: “sensory neuron” (2012–2020), “disease” (2013–2020), “mechanical channel” (2013–2020), and “protein” (2013–2019).

**Table 4.** The keywords with the strongest citation bursts of publications on Piezo researches

We conducted a bibliometric analysis of relevant Piezo channel research as well as a visual description of the research status and trends, which allowed a systematic understanding of the past and future via CiteSpace and VOSviewer.

### Overall information

By analyzing 431 collected articles, we calculated the growth trend over time. Our data suggested that the literature related to Piezo channels showed an increasing trend between 2010 and 2020. There is no doubt that the United States made an outstanding contribution to the field of Piezo research. However, China, England, and Germany have also played a key role in advancing this field.

According to the publication counts and centrality, institutions with strong scientific research standards are mainly concentrated in higher education research institutions, which are important bases for scientific research and education. The top 10 institutions published 104 articles, accounting for 25.61% of the total publications. In this list, seven originate from USA (The Scripps Research Institute, SUNY Buffalo, Novartis Research Foundation, Yale University, Duke University, Stanford University, University of Alabama at Birmingham). This indicates that the institutions originating from the USA are at the forefront in terms of absolute contribution and relative influence, which is consistent with the analysis of national contribution in the field of Piezo. In addition, the pharmaceutical company Novartis, located in the USA, published several relevant research studies [1,3,21,22].

Among the top 10 academic journals, six journals had an impact factor higher than 4. Of these, Nature (IF = 42.778, 2019) accounted for 1.74% of all publications. Nineteen journals (about 10% of the total) published 206 papers, accounting for 47.79% of the total. The average impact factor of these journals is 10.95, which means that nearly half of all Piezo-related articles were published in high-quality, high-impact journals. This indicates that the quality of related research in the field of Piezo channel is relatively high and should be maintained.

The USA maintained the dominant position in terms of publications, citation frequency, and H-index, suggesting that the US plays a crucial role in this field. Moreover, China plays an advantageous role in terms of publication numbers (76), although its citations (699) are the lowest in the top five countries. Encouragingly, China is the only developing country in the top five countries. Strong collaborations among countries can drive research, and in this case, strong collaborations were found between the US, China, England, Germany, and Japan. These collaborations increased the number of published papers, as well as the quantity and quality of research on Piezo channels.

## Discussion

### Intellectual base

The network of citations has recently become a building block for a mathematical, graph-based theory of networks in the informatics sciences. Highly cited articles were analyzed to determine the intellectual base in the field [23]. Citation of an article in another article is important for scientific publications and is representative of its quality. These include, but are not limited to, the quality of the work, overall interest in the topics discussed, and impact of an article on the future work of the researchers.

**Figure 6.**
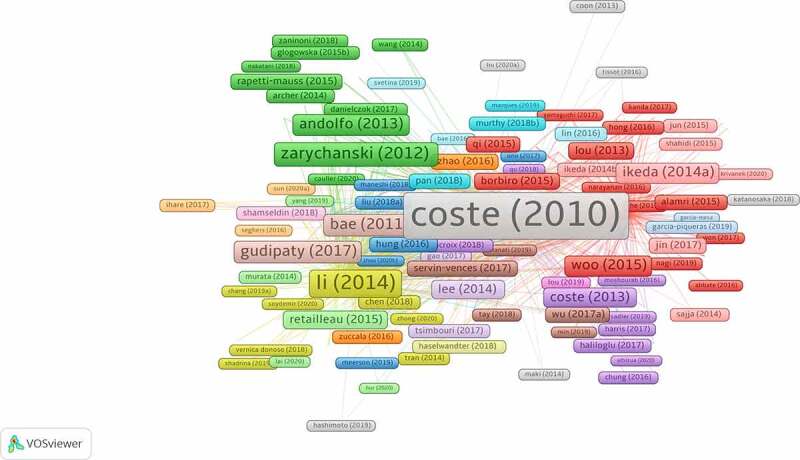
The citation network map of publications

From the article-citation network (Figure 6) on the Piezo channel over the past 10 years, Bertrand Coste’s article is the most-cited paper and is located in the center of the network. They revealed that Piezo serves as a component of mechanically activated cation channels, which is important for inducing mechanically activated cationic currents in cells [1]. As shown in Eisenhoffer’s study, mechanical stimulation induces live cell extrusion to maintain homeostatic cell numbers in the epithelia, and Piezo1 plays an important role in this process [24]. Bertrand Coste et al. demonstrated that Piezo proteins are pore-forming subunits of mechanically activated channels and are evolutionarily conserved ion channel families involved in mechanotransduction [21].

In hematologic systems, Piezo1 contributes to many physiological and pathophysiological processes via mechanotransduction. The first disease related to Piezo was demonstrated in 2012, and it was reported that mutations in Piezo1 are associated with hereditary xerocytosis [10,20]. In terms of hereditary xerocytosis, gain-of-function mutations in the Piezo1 channel are linked to this disease [25]. It is possible that increased Piezo1 activity directly increases the influx of Na^+^ and efflux of K^+^. Other mechanisms could indirectly account for this phenotype. The Piezo1-mediated increase in intracellular Ca^2+^ concentration could induce K^+^ efflux by activating the Gardos channel, which causes a reduction in erythrocyte cell volume [26]. In 2015, Cahalan’s study further illustrated the mechanism of Piezo in hereditary xerocytosis. Gain-of-function mutations of Piezo1 causes calcium influx and the subsequent dehydration of RBCs via downstream activation of the KCa3.1 Gardos channel, directly implicating Piezo1 in RBC volume control [27]. Moreover, Piezo1 channels are likely to function in normal RBCs and regulate ATP release via a previously unidentified mechanotransductive pathway [5]. Gain-of-function mutations in Piezo1 cause hereditary xerocytosis in humans, while the phenotypic consequence of Piezo1 loss of function in humans causes congenital lymphatic dysplasia [28].

Piezo1 has been shown to be expressed in tissues of the cardiovascular system [11]. It is involved in vascular development and cardiogenesis by mechanical stimulation owing to blood flow. Alternatively, Piezo1-dependent calcium influx could influence vascular remodeling by modulating multiple downstream signaling pathways. Piezo is not only a determinant of vascular architecture during early development but is also involved in the structural remodeling of small arteries. It has been shown to be involved in the remodeling of small arteries upon hypertension. This process is associated with a rise in cytosolic calcium mediated by Piezo1 and stimulates the activity of transglutaminases, which are cross-linking enzymes required for the remodeling of small arteries [29]. Another method by which Piezo regulates blood pressure was reported by Wang et al. [30]; arterial blood pressure is controlled by vasodilatory factors, such as nitric oxide (NO), released from the endothelium. In this process, the mechanosensitive cation channel Piezo1 is required for flow-induced ATP and NO release.

Piezo1 also senses the local cellular environment in neurons and other cells, thus promoting specific cell-cell interactions, cell differentiation, and cell motility [4,31,32].

In addition, the involvement of Piezo1 in cell motility may explain the relationship between the down-regulation of Piezo1-mediated activity in the breast cancer cell line MCF-7, resulting in decreased motility of MCF-7 cells [33].

Several tissue-specific conditional knockout lines have shown that Piezo2 mediates much of the organism’s response to light mechanical touch. Specifically, Piezo2 channels confer the mechanically sensitive current in Merkel cells; consistent with this, skin-specific knockout of Piezo2 leads to reduced light touch responses [3,34]. In addition, humans with autosomal recessively inherited loss-of-function variants in Piezo2 exhibited general losses in vibration detection, touch discrimination, and joint proprioception [35]. Mutations in Piezo2 are mainly associated with several arthrogryposis disorders [36,37]. Two of these mutations destabilize inactivation, resulting in an overall increase in calcium influx.

In general, Piezo channels are expressed in many types of cells and play an important role in a variety of physiological processes, from the regulation of red blood cell volume to gentle touch sensation, and are associated with many diseases.

### Research frontiers

The frontiers of Piezo research were predicted using the strongest citation bursts of publications. The top seven research frontiers of Piezo were currents, sensory neurons, diseases, mechanical channels, proteins, distal arthrogryposis, and mechanoreceptors.

Mechanical stimulation drives many physiological processes, including touch and pain, hearing, and blood pressure regulation. Mechanically activated (MA) cation channel activity has been recorded in many cells, but the responsible molecule has not been determined. One study showed that Piezo1 (Fam38A) was necessary for MA current in these cells [1]. Piezo is a pore-forming subunit that provides mechanically activated currents when expressed in heterologous systems and is necessary for many cellular mechanical responses [1,21,38,39]. In a sense, Piezo can be regarded as a real mechanically activated ion channel [40].

Piezo is a very large protein (human Piezo1 and human Piezo2 consist of 2521 and 2752 amino acids, respectively). Each subunit has many (> 14) predicted transmembrane (TM) domains. Surprisingly, it has no homology with other known proteins [1]. The overall shape of Piezo is a propeller-shaped trimer complex with three curved “blades” surrounding a central pore topped by a cap called the C-terminal extracellular domain [41].

Mechanotransduction is the process in which a mechanical stimulus is transduced into biological signals within a cell. Although this channel is highly expressed in a wide range of mechanically sensitive cells, the distribution of the two isoforms is different. Piezo2 is mainly present in sensory tissues such as the dorsal root ganglion (DRG), sensory neurons, and Merkel cells, which respond to touch [3]. Meanwhile, Piezo1 is mainly expressed in non-sensory tissues (such as kidneys and red blood cells) that are exposed to fluid pressure and flow [42]. This unique distribution pattern can also be seen in other species, such as Piezo1 in erythrocytes and Piezo2 in Rohon-Beard sensory neurons in zebrafish [24] and Piezo2 in trigeminal ganglion sensory neurons in star-nosed mole rats and birds [42–44]. This suggests that this distribution is conserved.

In vertebrates, the expression of Piezo channels is both necessary for survival and is associated with many diseases. Global knockout of Piezo1 or Piezo2 in mice is lethal [11,22]. Functional mutations can disrupt mechanotransduction via Piezo. Mutations in Piezo have been linked to various human disorders. Gain-of-function mutations in Piezo1 are associated with dehydrated hereditary xerocytosis [10,20,25]. In contrast, the loss of function of Piezo1 in humans causes congenital lymphatic dysplasia [28]. Notably, there appears to be paradox because of the overlap in symptoms of these two disorders; some xerocytosis patients also have lymphedema [45], while erythrocytes in lymphedema patients show stomatocytes [46]. Mutations in Piezo2 are mainly associated with several arthrogryposis disorders [36,37].

In general, as a mechanotransduction molecule and mechanically activated ion channel, Piezo protein has revealed many unexpected functions other than mechanical transduction. However, further research in this domain is warranted. In particular, little is known about the exact mechanism of action of Piezo in mechanotransduction. In addition, the role of Piezo has not been explored in many mechanical transduction processes.

## Strengths and limitations

To the best of our knowledge, this study is the first bibliometric analysis of Piezo trends. We set no time limit for our literature retrieval, and the data downloaded from WOS covered the vast majority of articles in the field of Piezo research. The data analysis was more objective and comprehensive, clearly showing the current situation of Piezo research. However, the study entirely consisted of original articles published in 2010–2020 and included in the WOS database. Since books, conference abstracts, and other types of publications were not included in the literature screening, our data may not represent the literature in its entirety. In addition, only English articles were included in our analysis, which makes our analysis incomplete. In terms of retrieving the database, we only retrieved publications from the WOS database. While other databases such as PubMed, Scopus, and Embase provide broader coverage of the scientific literature, WOS CC is superior at providing detailed data (for example, annual publications, author information, journal sources, and national and institutional information). It is important to note that the results of this study are stable and reproducible. The study now covers the vast majority of papers from 2010 to 2020, and the newly published papers would not affect the final results.

## Conclusion

We have drawn scientific maps of a series of journals, countries, institutions, authors, co-citations, and citation burst keywords to determine the thematic evolution and trends of the knowledge map in this field. Piezo has broad development prospects because of its worthy and promising future. An appreciable number of papers have been published in influential journals. The United States dominates in this area. The Scripps Research Institute, SUNY Buffalo, Tsinghua University, Novartis Research Foundation, and Yale University are considered excellent institutions for research cooperation. Ardem Patapoutian is a prominent researcher in this field. Bibliometric analysis of the literature on Piezo is of great significance for researchers in determining cooperative relationships, discovering research hotspots, and predicting the frontiers of Piezo channel research.

## Geolocation information

Lanzhou, Gansu, China
